# Diabetes mellitus and hypertension increase the risk of colorectal cancer mortality; a robust Bayesian adjustment analysis 

**Published:** 2017

**Authors:** Maryam Nasserinejad, Ahmad Reza Baghestani, Sadjad Shojaee, Mohamad Amin Pourhoseingholi, Hadis Najafimehr, Mehrdad Haghazali

**Affiliations:** 1 *Department of Biostatistics, Faculty of Paramedical Sciences, Shahid Beheshti University of Medical Sciences, Tehran, Iran.*; 2 *Gastroenterology and Liver Diseases Research Center, Research Institute for Gastroenterology and Liver Diseases, Shahid Beheshti University of Medical Sciences, Tehran, Iran *; 3 *Basic and Molecular Epidemiology of Gastrointestinal Disorders Research Center, Research Institute for Gastroenterology and Liver Diseases, Shahid Beheshti University of Medical Sciences, Tehran, Iran.*; 4 *Behbood Gastroenterology and Liver Diseases Research Center, Shahid Beheshti University of Medical Sciences, Tehran, Iran*

**Keywords:** Colorectal cancer, Diabetes, Hypertension, Misclassification, Bayesian analysis

## Abstract

**Aim:**

The aim of this study was to investigate the impact of diabetes and hypertension on colorectal cancer (CRC) mortality.

**Background:**

One of the methodology in epidemiological studies is to use self-report questionnaires to gather data, this is the easiest and cheapest method but involves with misclassification bias. We use robust Bayesian adjustment to correct this bias.

**Methods:**

One of the methodology in epidemiological studies is to use self-report questionnaires to gather data, this is the easiest and cheapest method but involves with misclassification bias. We use robust Bayesian adjustment to correct this bias.

**Results:**

The effect size with ignorance misclassification bias was 0.78 for diabetes and 0.94 for hypertension respectively which both of them were not significant. After adjusting the misclassification and performing the robust Bayesian analysis, we arrived at region (0.27, 3.4) for OR of diabetes and (0.21, 2.31) for hypertension.

**Conclusion:**

our study demonstrated that diabetes and hypertension increase the risk of mortality in CRC patients, using robust Bayesian analysis and misclassification in diagnosis these two exposure could change or confound the results of this association.

## Introduction

 The prevalence of diabetes is rapidly rising all over the globe at an alarming rate. It has been estimated that the global burden of type II diabetes mellitus will be increases up to 592 million in 2035, which is a 55% increasing compared to 2013 (1). A systematic review estimated that there were five million deaths attributed to diabetes in 2015 and the number of people with diabetes was predicted to rise to 642 million by 2040 (2). The prevalence of type II diabetes among Iranian older adults was estimated 14.4% at the community level (3). Also for hypertension, a systematic review of 90 countries identified that 31.1% of the world's adults had hypertension (4) and the prevalence of hypertension was estimated 26.6% in Iran (5).

On the other hand, because of the low level of awareness about two diseases and lack of symptoms, many people are unaware about diabetes and hypertension, even though, they have these two diseases and this lack of knowledge leads to misclassification. For instance in Iran, prevalence of diagnosed and undiagnosed diabetes were 8.1%, 5.1% in men and 10%, 4.7% in women respectively (6) and the prevalence of hypertension was estimated 20.1, while the self-reported hypertension was 12.3 (7). These differences are due to people's lack of knowledge of their illness. 

Recent studies indicate that diabetes and hypertension can increase the risk of cancer or the risk of death from cancer (8, 9). 

In our data, two variables, hypertension and diabetes mellitus that are considered as exposures of mortality due to colorectal cancer (CRC) were registered in the medical file of the person by self-report. Considering that the self-reported in the two diseases mentioned is less than the actual level, so some people with these two diseases are classified as non-exposure. In fact, the sensitivity of self-reported is less than one. On the other hand, it is unlikely that a person who is healthy, declares that he had diabetes (or hypertension) and was classified into exposure group. So the specificity of self-report is close to one. According to the above explanations, one-way misclassification error occurs in the data that results in under-reporting of the exposures, Therefore, the estimated of Odds ratios for this data will not be reliable.

The goal of this study is to use Robust Bayesian analysis for adjustment odds ratio for two exposures (hypertension and diabetes) of mortality in colorectal cancer. 

## Methods

We used data from a retrospective cohort study, conducted on colorectal cancer patients who registered in Gastroenterology and Liver Disease center research at Taleqani hospital, Tehran from 2001 to 2010 (10, 11). 236 mortality cases selected from databank of this mentioned cohort and 889 colorectal cancer patient who have not been experienced the death up to 2010 as the controls, which adjusted by age and sex (12). 

**Table 1:** Impact of Diabetes and Hypertension of colorectal mortality

Variable		case	control	Odds ratio (95% CI)	p-value
Diabetes	+	12	65	0.78 (0.41 to 1.47)	0.43
	-	155	652		
	Total	167	717		
Hypertension	+	19	96	0.94 (0.49 to 1.39)	0.47
	-	145	605		
	Total	164	701		

Diabetes and hypertension were considered as exposures. According to the explanations and given that the present study is retrospective, there is a high risk for misclassification of two exposures. To adjust the misclassified exposure and then correct the effect size (OR), we should get feasible ranges for sensitivity and specificity of self-report and true exposure prevalence of diabetes and hypertension in patients with cancer. The average age of patients in this study was 53.59 and according to previous studies, prevalence of diabetes in Iranian elderly was estimated 24.5 (13) so for feasible range of true exposure prevalence, we chose (0.2, 0.3), and for hypertension by considering that hypertension in Iranian elderly people was 68% (14) we chose the range (0.63, 0.73).

For sensitivity and specificity of self-report in diabetes, we chose (0.1, 0.4) and (0.9, 1) and for hypertension, we chose the ranges (0.1, 0.3) and (0.9, 1), respectively. θ is consider to be the log odds ratio. To perform robust Bayesian analysis (15), normal prior distribution with the mean of 0 and the variance of 0.5 was assumed for the θ in both exposures. The choice of this prior distribution for θ is completely impartial. We conducted all analyses using R software, version 3.3.2 and Matlab software. 

## Results

The study included 1125 CRC patients who divided into two case and control groups. The mean age in the case and control were 53.56 and 53.57 years respectively. There was no significant difference between the mean age of case and control (P = 0.994). In case group, 150 patients were male and 89 were female and in controls 539 patients were male and 350 were female, It is worth mentioning that there was no difference between gender (P= 0.456), which means that controls were matched for age and gender. In case and control groups 12 (7.2%) and 65 (9%) patients had diabetes and 19 (11.6%) and 96 (13.7%) had hypertension respectively, according to patients’ records and their self-report (Table 1). 

Without adjusting for misclassification, both Diabetes and hypertension have no effect on risk of colorectal cancer mortality (OR 0.78, 95% CI 0.41- 1.47) and (OR 0.94, 95% CI 0.49- 1.39). But with considering the misclassification probabilities and using robust Bayesian approach, due to the selected ranges for sensitivity, specificity, true exposure prevalence and prior distribution, we arrived at region (-1.3, 1.24) for log OR and (0.27, 3.4) for OR with diabetes as exposure and given the hypertension as exposure, we arrived at the (-1.52, 0.78) for log OR and (0.21, 2.18) for OR, Where every point of this regions can be reported, the most neutral of which is the middle of the regions, so points 1.84 and 1.2 are considered as OR with diabetes and hypertension as exposure, respectively.

## Discussion

The present study indicated that the misclassification could confound the estimated OR as the result of association between exposure and outcome. The OR obtained from the analysis without adjusting the misclassification was not significant for both diabetes and hypertension. This means, there is no association between diabetes mellitus, hypertension and risk of colorectal cancer mortality, while the results of the robust Bayesian analysis are completely different, it showed that diabetes and hypertension have important role in mortality due to colorectal cancer. The odds ratio is interpreted that the risk of mortality for CRC patients with diabetes was 1.84 times more than those without diabetes. For hypertension, point 1.2 is middle of region for estimated OR which means the hypertension increased the risk of mortality 1.2 times more, compared to CRC patients without hypertension. 

This study is a first study to investigate the effect of diabetes and hypertension on mortality due to colorectal cancer with adjusting misclassified exposures. Similar studies which aimed to investigate the relationship between diabetes and CRC, just using cox proportional hazard model, which makes it impossible to adjust misclassification error. In the study of Minlikeeva et al (16) investigated the effects of hypertension, diabetes and heart disease on ovarian cancer, they used the proportion hazard regression model, their effect size was HR, which results about diabetes, was consistent with our results, but did not find significant relation with hypertension. This difference may be due to ignoring the probability of misclassification in the data or difference in the type of cancer. Harding et al (17) investigated only the effect of hypertension on cancer incidence and cancer mortality, their results indicated that the treated hypertension did not increase the risk of cancer incidence and mortality compared with untreated hypertension. A 2002 meta-analysis found 23% increased risk for mortality in those with the highest grade of hypertension in comparison with lowest (18), which their result is very close to the our study. Ce Tan et al. (19) and Bella et al. (20) conducted similar studies with the same results; they found that diabetes was associated with colorectal cancer death, increasing the risk of mortality up to 40% and 36%, respectively. In study by Walker et al. (21), they reported HR = 1.57 for men and HR = 1.84 for women, regarding the association of diabetes and risk of mortality. They considered type 2 diabetes as an exposure. Although their results were similar to ours, we achieved a significant impact with the adjustment of misclassification not only for diabetes, but also for hypertension. 

In conclusion, our study demonstrated that diabetes and hypertension increase the risk of mortality in CRC patients, using robust Bayesian analysis and misclassification in diagnosis these two exposure could change or confound the results of this association.
